# Acute Corticotropin-Releasing Factor Receptor Type 2 Agonism Results in Sustained Symptom Improvement in Myalgic Encephalomyelitis/Chronic Fatigue Syndrome

**DOI:** 10.3389/fnsys.2021.698240

**Published:** 2021-09-01

**Authors:** Gerard Pereira, Hunter Gillies, Sanjay Chanda, Michael Corbett, Suzanne D. Vernon, Tina Milani, Lucinda Bateman

**Affiliations:** ^1^Cortene Inc., Burlingame, CA, United States; ^2^Bateman Horne Center, Salt Lake City, UT, United States

**Keywords:** myalgic encephalomyelitis chronic fatigue syndrome, ME/CFS, agonist-mediated receptor endocytosis, corticotropin-releasing factor receptor 2, CRF2, serotonin, homeostasis

## Abstract

**Background:**

Myalgic encephalomyelitis/chronic fatigue syndrome (ME/CFS) is a complex multi-symptom disease with widespread evidence of disrupted systems. The authors hypothesize that it is caused by the upregulation of the corticotropin-releasing factor receptor type 2 (CRFR2) in the raphé nuclei and limbic system, which impairs the ability to maintain homeostasis. The authors propose utilizing agonist-mediated receptor endocytosis to downregulate CRFR2.

**Materials and Methods:**

This open-label trial tested the safety, tolerability and efficacy of an *acute* dose of CT38s (a short-lived, CRFR2-selective agonist, with no known off-target activity) in 14 ME/CFS patients. CT38s was subcutaneously-infused at one of four dose-levels (i.e., infusion rates of 0.01, 0.03, 0.06, and 0.20 μg/kg/h), for a maximum of 10.5 h. Effect was measured as the pre-/post-treatment change in the mean 28-day total daily symptom score (TDSS), which aggregated 13 individual patient-reported symptoms.

**Results:**

ME/CFS patients were significantly more sensitive to the transient hemodynamic effects of CRFR2 stimulation than healthy subjects in a prior trial, supporting the hypothesized CRFR2 upregulation. Adverse events were generally mild, resolved without intervention, and difficult to distinguish from ME/CFS symptoms, supporting a CRFR2 role in the disease. The acute dose of CT38s was associated with an improvement in mean TDSS that was sustained (over at least 28 days post-treatment) and correlated with both total exposure and pre-treatment symptom severity. At an infusion rate of 0.03 μg/kg/h, mean TDSS improved by −7.5 ± 1.9 (or −25.7%, *p* = 0.009), with all monitored symptoms improving.

**Conclusion:**

The trial supports the hypothesis that CRFR2 is upregulated in ME/CFS, and that acute CRFR2 agonism may be a viable treatment approach warranting further study.

**Clinical Trial Registration:**

ClinicalTrials.gov, identifier NCT03613129.

## Introduction

### Myalgic Encephalomyelitis/Chronic Fatigue Syndrome

Myalgic encephalomyelitis/chronic fatigue syndrome (ME/CFS) is a debilitating disease affecting ∼20 million worldwide. It can be triggered by infection, traumatic life events, chemicals, toxins, immunizations, anesthetics, physical trauma, among others (Chu et al., 2019). Its major symptoms include profound fatigue (described as “concrete limbs,” “crushing gravity,” “not tired”), musculoskeletal pain, cognitive impairment (“brain fog”), orthostatic intolerance (OI), flu-like symptoms and un-refreshing sleep (Chu et al., 2018, 2019). These are exacerbated by any kind of stimulation, including physical, cognitive, emotional (Chu et al., 2018) and even postural change (van Campen et al., 2021), referred to as post-exertional malaise (PEM). ME/CFS patients often have other disorders, including dysautonomia (Naschitz et al., 2004; Newton et al., 2007), insulin resistance (Maloney et al., 2010; Armstrong et al., 2015), immune dysfunction (Straus et al., 1988; Borish et al., 1998; Nicolson et al., 2002; Chia and Chia, 2007; Morris et al., 2014), hypothyroidism (Ruiz-Núñez et al., 2018) and gynecological issues (Boneva et al., 2015). ME/CFS is diagnosed by exclusion, treated symptomatically and results in a quality of life lower than in most chronic diseases (Hvidberg et al., 2015). Research has identified abnormalities in neurotransmitters, hormones, autoantibodies, immune cells, cytokines, metabolites, energy substrates, oxidative byproducts, mitochondria, ion channels, gut bacteria, genetics, epigenetics, and brain anatomy, but to date, no single cause explains this dysfunction (Komaroff, 2019).

### Etiological Hypothesis

**FIGURE 1:**
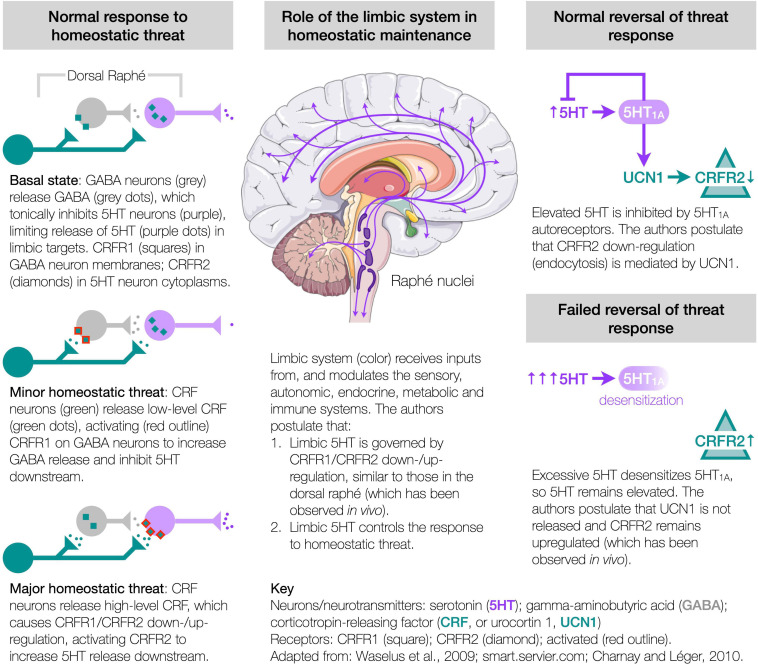
Etiological hypothesis.

The authors hypothesize that these abnormalities could all originate from a single pathway, involving the corticotropin-releasing factor (CRF, also known as corticotropin-releasing hormone or CRH) system and its regulation of serotonin (5HT) in the limbic system, which controls the response to homeostatic threat (Dedic et al., 2018; Deussing and Chen, 2018; Godoy et al., 2018), usually termed “stress,” but avoided here to preclude narrow connotations. This hypothesis synthesizes numerous independent *in vivo* studies to propose the mechanisms of homeostasis. It rests on three constructs (Figure 1).

*First, under homeostatic threat, the CRF system controls the release of 5HT from the dorsal raphé*. Within the dorsal raphé (largest of the raphé nuclei and a major source of brain 5HT), CRF receptor type 1 (CRFR1) is present in the membranes of gamma-aminobutyric acid (GABA) neurons, while CRFR2 is present in the cytoplasm of 5HT neurons—this configuration being associated with a basal level of 5HT output (Waselus et al., 2005; Kirby et al., 2008; Lukkes et al., 2008). Minor homeostatic threat, releasing low levels of CRF in the dorsal raphé, activates CRFR1 to release GABA, which tonically inhibits 5HT downstream (Waselus et al., 2005; Kirby et al., 2008; Lukkes et al., 2008). Major (or prolonged/repeated) threat, releasing high levels of CRF, causes the CRF receptors to redistribute, with CRFR1 internalizing (in GABA neurons), and CRFR2 migrating to the membranes of 5HT neurons (Waselus et al., 2009), where activation releases 5HT downstream (Waselus et al., 2005; Kirby et al., 2008; Lukkes et al., 2008). Thus, the CRF system controls the threat-specific release of 5HT from the dorsal raphé (and possibly other raphé nuclei).

*Second, homeostatic threat induces threat- and neuronally-specific adaptations of CRFR1 and CRFR2 to control limbic 5HT and homeostasis*. The limbic system receives threat-related inputs from the sensory (Chanes and Barrett, 2016), autonomic (Berthoud and Neuhuber, 2000), endocrine (Dedic et al., 2018; Deussing and Chen, 2018), metabolic (Tups et al., 2017; Pozo and Claret, 2018) and immune (Silverman et al., 2005; Soto-Tinoco et al., 2016; Dantzer, 2018; Kipnis, 2018) systems. The authors propose that these inputs engage the CRF system (Dedic et al., 2018; Deussing and Chen, 2018), inducing adaptations in CRFR1/CRFR2 similar to those in the dorsal raphé and observed *in vivo*, e.g., in the hippocampus (Sananbenesi et al., 2003) or amygdala (Qi et al., 2014), which then modulate 5HT (Hensler, 2006; Charnay and Léger, 2010) and other neuromodulators (Avery and Krichmar, 2017). That is, an initial CRFR1/CRFR2 configuration associated with a basal 5HT output (Waselus et al., 2005; Kirby et al., 2008; Lukkes et al., 2008), similar to a homeostatic set point, adapts by upregulating either CRFR1 or CRFR2 (concomitant with downregulating CRFR1), respectively, decreasing or increasing 5HT to control downstream function. This enables a coordinated threat response of 5HT-related functions, including emotion, motivation, nociception, memory consolidation, regulating extrapyramidal and ventricular systems (Berger et al., 2009; Waselus et al., 2011), motor control (Perrier et al., 2013; Perrier and Cotel, 2015), proprioception (Delhaye et al., 2018), sensory sensitivity (Heath et al., 2006; Petzold et al., 2009; Hurley and Hall, 2011; Obara et al., 2014; Chang et al., 2016; Shimegi et al., 2016), respiration (Hilaire et al., 2010), thermoregulation (Lin et al., 1998; Boulant, 2000), and downstream autonomic, endocrine (Dedic et al., 2018; Deussing and Chen, 2018; Godoy et al., 2018), metabolic (Donovan and Tecott, 2013; Roh et al., 2016; Tups et al., 2017; Pozo and Claret, 2018; Flak et al., 2020; Alonge et al., 2021), and immune (Quintanar and Guzmán-Soto, 2013; Sundman and Olofsson, 2014; Soto-Tinoco et al., 2016; Dantzer, 2018) actions. Importantly, CRFR1/CRFR2 adaptations are both threat- and neuronally-specific. For instance, a high temperature *must* elevate 5HT in the neurons of the preoptic area of the anterior hypothalamus to provoke cooling (Lin et al., 1998; Boulant, 2000), but without modulating other 5HT neurons, e.g., vision/hearing-related. In contrast, the selective serotonin reuptake inhibitors (SSRIs) indiscriminately modulate 5HT, so can cause fever, chills, blurry vision, tinnitus, etc. (Ferguson, 2001). Thus, threat- and neuronally-specific CRFR1/CRFR2 adaptions control limbic 5HT and downstream function.

*Third, CRFR2 (and CRFR1) are susceptible to maladaptation*. Following threat resolution, basal configurations are restored, with 5HT_1A_ autoreceptors inhibiting further 5HT release (Neufeld-Cohen et al., 2012), and CRFR2 downregulating *via* endocytosis (Markovic et al., 2008, 2011; Hauger et al., 2013), likely mediated by urocortin 1 (UCN1) (Dos Santos et al., 2015) competitively displacing CRF—respective CRFR2 binding affinities are 0.4 and 44.5 nmol (Deussing and Chen, 2018). However, excessive 5HT can desensitize the 5HT_1A_ autoreceptors leaving 5HT elevated (Rozeske et al., 2011), which the authors propose disrupts the restorative process and fails to downregulate CRFR2 at threat cessation, observed *in vivo* as increases in CRFR2 expression (Lukkes et al., 2009), or increased membrane-bound CRFR2 in the dorsal raphé (Waselus et al., 2009; Wood et al., 2013), hippocampus (Sananbenesi et al., 2003), amygdala (coincident with decreased CRFR1 expression) (Qi et al., 2014), and bed nucleus of the stria terminalis (BNST) (Lebow et al., 2012), yet absent in the BNST under different provocation (Elharrar et al., 2013), so supporting the threat-specificity of CRFR1/CRFR2 adaptation. Such CRFR2 upregulations can accumulate and bias the system toward elevated 5HT release (Lukkes et al., 2009; Wood et al., 2013). ME/CFS develops more in conditions of adversity (Heim et al., 2006; Nater et al., 2011), and patients show evidence of overactive limbic circuits (Nakatomi et al., 2014), elevated brain 5HT (Bakheit et al., 1992; Dinan et al., 1997; Sharpe et al., 1997), 5HT_1A_ desensitization throughout the limbic system (Cleare et al., 2005), and numerous symptoms indicative of an inability to control the many functions modulated by 5HT (e.g., fatigue, proprioception, dyspnea, sensory sensitivity, dysautonomia, hypothyroidism, glucose control, immune function). Many signs, symptoms and anomalies of ME/CFS have been linked to 5HT (and downstream mediators), but studies involving SSRIs require cautious interpretation as these drugs *initially increase* extracellular 5HT, before activating the 5HT_1A_ autoreceptors to decrease 5HT and induce effect (Andrews et al., 2015). **Thus, the authors hypothesize that ME/CFS results from CRFR2 upregulations in specific neurons of the raphé nuclei and limbic system, leading to a loss of homeostatic control over the functions mediated by those neurons**.

### Therapeutic Approach

If upregulated CRFR2 causes ME/CFS, the resulting inability to maintain homeostasis under *dynamic* threat cannot be repaired by *static* approaches (e.g., fixed-doses of CRF/5HT antibodies, CRFR2 antagonists or GABA agonists). This leaves CRFR2 downregulation as the most reasonable approach.

*In vivo*, subcutaneous CT38s, a short-lived CRFR2-selective agonist with no known off-target activity (Supplementary Material), induces dose-dependent changes in norepinephrine and corticosterone release, spontaneous movement (possibly motor effect), gastrointestinal transit, urine volume, respiratory minute volume, core body temperature, heart rate (HR) and mean arterial pressure (MAP) (Supplementary Figures 1A–I). That is, CRFR2 stimulation in *healthy* rats produces signs analogous to complaints of ME/CFS. These data also suggest that CT38s enters the central nervous system as: norepinephrine/corticosterone involve the hypothalamus (Dedic et al., 2018; Deussing and Chen, 2018; Godoy et al., 2018); respiration and core body temperature involve 5HT in the medullary respiratory neurons (Hilaire et al., 2010) and preoptic area of the anterior hypothalamus (Lin et al., 1998; Boulant, 2000), and consistent with CRFR2 stimulation elevating 5HT to decrease respiratory function and temperature (Supplementary Figures 1F–G); and HR involves CRFR1 and CRFR2 in the BNST (Oliveira et al., 2015). Escalating doses cause the HR and MAP responses to peak and then decrease, notably at lower concentrations or exposures by infusion than by bolus (Supplementary Figures 2A–D). Since agonist-mediated receptor endocytosis increases with agonist concentration and the duration of stimulation (Markovic et al., 2008, 2011; Hauger et al., 2013), this apparent loss of HR and MAP sensitivity plausibly resulted from CRFR2 endocytosis in limbic neurons, occurring at lower concentrations by infusion due to the additive effect of time. **Thus, the authors propose utilizing agonist-mediated CRFR2 endocytosis to treat ME/CFS**.

## Materials and Methods

### InTiME

InTiME **In**vestigated the safety and efficacy of subcutaneously-dosed C**T**38s **i**n **ME**/CFS patients. This open-label trial was conducted at the Bateman Horne Center, under a physician-sponsored investigational new drug application filed with the United States Food and Drug Administration (FDA), registered at ClinicalTrials.gov (NCT03613129), approved by Aspire IRB, in compliance with the Declaration of Helsinki and Good Clinical Practice, with all patients providing informed consent.

### Aims

**FIGURE 2:**
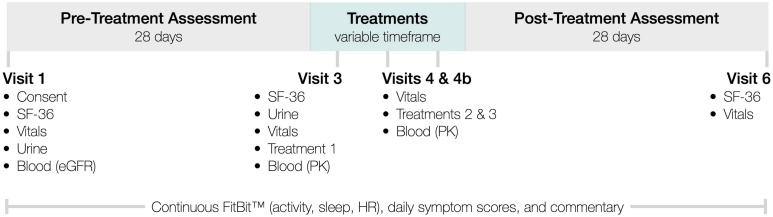
InTiME schema.

InTiME sought to show that an *acute* dose of subcutaneous CT38s in ME/CFS patients, could safely induce sustained symptom improvement, determined by comparing symptoms (see below) in the 28-day pre-treatment assessment period and the 28-day post-treatment assessment period (Figure 2). CRFR2 expression in the raphé nuclei and limbic system is neuronally-specific and adapts in realtime so cannot be measured. However, the observation of dose-dependent effect would link CRFR2 with ME/CFS, as CT38 is CRFR2-selective and has no off-target activity. Sustained effect would suggest CRFR2 endocytosis had occurred, as CT38 does not persist in rats or healthy humans, evidenced by the rapid normalization of induced HR increases (Supplementary Material), which are CRFR1/CRFR2-mediated in the BNST (Oliveira et al., 2015).

### Patients

InTiME included 18–60 year-old, male or female patients, meeting the Fukuda, Canadian and National Academy of Medicine criteria for ME/CFS, living between 3,500 and 5,500 feet above sea level (related to cardio-pulmonary exercise testing, CPET), with a stable state of illness in the prior 3 months, i.e., absence of active or uncontrolled co-morbidities including infections or depression. InTiME excluded patients with untreated endocrine diagnoses, tachycardia, severe hypotension, renal impairment, or who were taking anti-retrovirals, short-term antivirals/antibiotics, rituximab, or medications interfering with 5HT, norepinephrine, dopamine or cortisol.

### Intervention

The drug substance, CT38s, is the acetate salt of CT38 (free base), a custom, 40-amino acid peptide. It is a potent CRFR2-selective agonist (EC50 nmol/% of Emax: 17.1/100), with no known off-target activity. The drug product was supplied in active (CT38s: 1 mg/ml) and diluent (vehicle: 0.05 M TRIS buffer, 0.67% NaCl in sterile H_2_O for injection, USP, pH 7.5-7.7) vials, requiring on-site dilution. The drug was delivered subcutaneously *via* programmable syringe pump (McKinley^TM^ T34) utilizing a soft cannula infusion set (Neria^TM^ Soft 90). Pharmacokinetic (PK) data are expressed in terms of CT38. CT38s has been studied in animals and healthy human subjects in a prior Phase 1 clinical trial (Supplementary Material), but data is not publicly available as it is part of an ongoing drug development program.

### Dosing

The *acute* dose was intended to reproduce the reduced HR sensitivity noted in healthy rats by infusion, where apparent endocytosis occurred at a total exposure (i.e., area under the plasma concentration-time curve or AUC) of ∼40 ng h/ml and a plasma concentration of at least ∼1.50 ng/ml, human equivalents of 7 ng h/ml and 1.40 ng/ml, respectively (Supplementary Figure 2). In an infusion, the maximum plasma concentration (Cmax) and AUC are governed by the rate and duration of infusion. Thus, the starting dose-level was planned as a 3-h treatment, involving a priming bolus of 0.15 μg/kg, and a continuous infusion at a rate (in μg/kg/h) of 0.20 for 45 min, escalating to 0.22 for 45 min, then escalating to 0.24 for 90 min—dose: 0.825 μg/kg (below the maximum tolerated *bolus* dose in the prior Phase 1 trial of 0.833 μg/kg, Supplementary Material); projected Cmax: 1.37 ng/ml (below the maximum tolerated concentration of 1.56 ng/ml in the prior Phase 1 trial, Supplementary Material); projected AUC: 4.16 ng h/ml. This was to be repeated at a second treatment to provide a total AUC of 8.33 ng h/ml. It was assumed that any achieved CRFR2 endocytosis would persist, so the number of treatments and their separation were not critical (generally planned as two treatments separated by at least two days, but varied due to dosing changes, see below). Assuming safety at this starting dose-level, InTiME planned two higher dose-levels in subsequent patients.

### Dosing Changes

**FIGURE 3:**
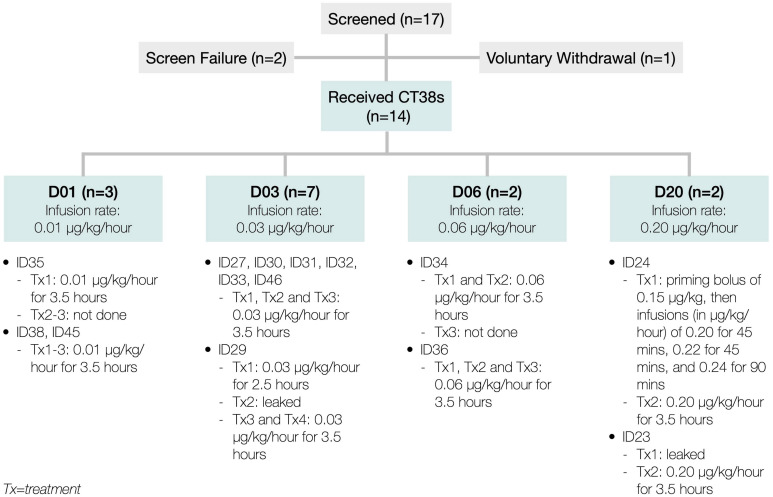
Patient disposition.

At the first treatment, the patient (ID24) experienced higher than anticipated hemodynamic changes. Since these changes were Cmax-related in rats and healthy subjects (Supplementary Material), the priming bolus and infusion rate escalations were eliminated, and the infusion duration increased to 3.5 h (referred to as D20, meaning 0.20 μg/kg/h), then utilized for ID24’s second treatment and both of ID23’s treatments. Both patients exhibited poor tolerability to D20. This necessitated reducing the dose-level to D03 (0.03 μg/kg/h) to lower the Cmax and adding a third treatment to increase the total AUC. To characterize the dose-response more fully, two additional dose-levels, D06 and D01, were also tested (Figure 3)—all approved by Aspire IRB.

### Blind Dose

Though open-label, patients were unaware of their relative doses. The first two patients (D20) expected the lowest dose, but received the highest. The next three patients (D03) knew that for safety reasons their dose was lower than the first two patients. The remaining patients did not know their relative dose-levels, and these were not administered sequentially. Patients had no contact with one another.

### Outcomes

The primary endpoint was the change in the mean total daily symptom score (TDSS), averaged over 28 days before the first treatment (TDSS_*pre*_) and 28 days before exit from the trial (TDSS_*post*_). The TDSS summed 13 individual symptoms (specifically, fatigue, muscle/joint pain, sleep issues, cognitive impairment, OI, abnormal temperature sensations, flu-like symptoms, headaches or sensory sensitivities, shortness of breath, gastrointestinal function, urogenital function, anxiety and depression), each patient-reported daily throughout the trial, on a 0–5 scale (0 = none, 1 = very mild, 2 = mild, 3 = moderate, 4 = severe, and 5 = very severe). The TDSS modifies the CFS Symptom Inventory (Wagner et al., 2005), expanding some symptoms (e.g., “sensory sensitivities” instead of “sensitivity to light”), collapsing others (e.g.,“abnormal temperature sensations” instead of “fever” and “chills”) and utilizing a single 24-h score (instead of intensity and frequency, considered too cumbersome for daily use).

Secondary endpoints included: (i) general health, assessed *via* the 36-Item Short Form Survey Instrument (SF-36, 0–100 scale, 0 = maximum disability, 100 = no disability) (Ware et al., 1994), completed at enrollment, prior to the start of treatment, and at exit, each referencing the preceding 4 weeks; (ii) Fitbit^TM^ metrics (activity, HR, and sleep), continuously monitored throughout the trial; and (iii) patient-reported daily assessments of: (a) completion of activities of daily living (ADL, 0 = not at all, 1 = a little, 2 = some, 3 = a lot, and 4 = completely); (b) avoidance of physical/mental exertion over PEM concerns (same as ADL scale); and (c) perceived level of physical/mental exertion in the prior 24 h (same as individual symptom scale). The original protocol included daily cognitive testing and CPET (both pre- and post-treatment). Both were eliminated, as the former showed evidence of patient learning before treatment, and the latter because seven patients had already undergone their post-treatment CPET before the third treatment was approved (i.e., after receiving only a fraction of the target AUC).

Safety measures included a blood test at enrollment (for blood chemistry, complete blood count and estimated glomerular filtration rate), and urine tests at enrollment and before treatment (for illicit drugs and pregnancy). During treatment, HR, systolic (sBP) and diastolic (dBP) blood pressure were recorded just before dosing (baseline), every 15-min thereafter, and for at least 90 min post-treatment. Dosing was to be stopped in a given patient, if HR > 120 bpm (or < 45 bpm), sBP < 90 mmHg or dBP < 50 mmHg; or if sBP or dBP decreased by more than 20 mmHg or 15 mmHg from baseline on three consecutive readings, respectively. PK blood samples were obtained at intervals before, during and after treatment. The principal investigator (PI) and site staff were responsible for soliciting, recording and reporting events qualifying as adverse events (AEs) and serious adverse events (SAEs), which were followed until resolution/stabilization.

### Statistics

InTiME was an exploratory study. All data are reported as mean and standard deviation, unless otherwise noted. Pre-/post-treatment data were compared by Student’s *t*-test. Relative sensitivity to CT38 among patients and healthy subjects were assessed by Kolmogorov-Smirnov test. Correlations were assessed by the Pearson product-moment correlation coefficient. No correction was applied for missing data, which was minimal (93.5% compliance). Study data were collected and managed using REDCap^®^ electronic data capture tools (Harris et al., 2009, 2019). Data collection was completed in April 2019.

## Results

### Patient Disposition

Between July 2018 and April 2019, 17 patients were consented and enrolled. Of these, two were screen failures, one voluntarily withdrew, and 14 received CT38 treatment at one of four dose-levels (Figure 3).

There were no study discontinuations. Two patients discontinued study drug, but remained in the study until exit: (i) ID35 (D01) received the first treatment, but experienced headache, facial numbness, body flushing, dyspnea, dizziness and swollen lymph nodes in the days following, so the PI decided to avoid further treatment; and (ii) ID34 (D06) received two treatments, but was noted to have poor venous access (necessary for blood sampling and safety, in the event of hypotension), so the PI decided to forego the third treatment.

There were three dose-related protocol deviations. ID23’s first treatment and ID29’s second treatment leaked at the cannula (observed by site staff and confirmed by PK). In addition, ID29’s first treatment was only 2.5 h (daylight savings time error), so ID29 was given a fourth treatment.

### Patient Demographics

The trial population was reasonably represented in sex (six male, eight female), age (mean: 43.9 years, range: 29.4–59.7 years), disease onset (eight gradual, six sudden), triggers (13 infectious, 4 toxins, 2 over-exertion, 4 emotional—some patients recorded multiple triggers), and disease duration (mean: 13.0 years, range: 2.1–25.0 years) (Table 1). Patients’ symptoms were heterogeneous (with 6 of 13 individual symptoms indicated as the worst, and 11 indicated among the worst 3), and of mild to moderate severity (TDSS_*pre*_ range: 13.8 to 44.7, Table 2).

**TABLE 1:** Patient demographics.

	ALL	D01	D03	D06	D20
*N*	14	3	7	2	2
Sex (Male/Female)	6/8	2/1	2/5	0/2	2/0
Race_White	12	3	5	2	2
Race_Other	2	0	2	0	0
Age (years)	43.7 ± 9.7	46.0 ± 8.2	39.7 ± 7.2	53.6 ± 0.6	44.6 ± 21.4
Age_Onset (years)	30.8 ± 12.7	31.0 ± 15.1	28.6 ± 12.4	41.5 ± 9.2	27.5 ± 19.1
Age_Diagnosis (years)	34.6 ± 12.3	33.7 ± 12.2	32.4 ± 10.4	46.0 ± 9.9	32.5 ± 24.7
Onset (gradual/sudden)	8/6	2/1	3/4	1/1	2/0
Triggers					
Infection	13	3	6	2	2
Toxins	4	1	2	0	1
Over-exertion	2	0	1	1	0
Emotional	4	0	3	0	1

*Age, Age_Onset and Age_Diagnosis indicate mean ± standard deviation.*

**TABLE 2:** CT38s dose, pharmacokinetics, mean TDSS and activity, by patient.

ID	Dose Group	Dose μg/kg	Mean Cmax ng/ml	Total AUC ng⋅h/ml	Mean TDSS ± σ		ΔTDSS (*p*-value)	Mean ΔADL (*p*-value)	Mean ΔPEM (*p*-value)	Mean ΔExertion (*p*-value)	Mean ΔSteps (*p*-value)
					TDSS_*pre*_	TDSS_*post*_					
ALL					29.5 ± 3.7	25.3 ± 3.7	−4.3 ± 1.4(0.011)	0.18 (0.04)	−0.17(0.10)	−0.24(0.05)	−262(0.40)
35	D01	0.035	0.12	0.59	32.4 ± 3.9	30.2 ± 3.4	−2.2 ± 1.0(0.038)	0.18 (0.11)	−0.18(0.11)	0.11 (0.37)	
38	D01	0.105	0.09	1.29	38.6 ± 3.3	32.8 ± 5.4	−5.8 ± 1.2 (< 0.001)	0.20 (0.34)	−0.23(0.24)	0.08 (0.64)	−100(0.81)
45	D01	0.105	0.11	1.02	15.0 ± 2.9	13.5 ± 3.1	−1.6 ± 0.8(0.031)	−0.32(0.16)	0.25 (0.28)	0.21 (0.44)	−115(0.61)
27	D03	0.315	0.24	3.44	24.9 ± 3.6	14.2 ± 3.9	−10.8 ± 1.1 (< 0.001)	0.13 (0.34)	0.12 (0.59)	−0.46(0.04)	−1,620(0.11)
29	D03	0.285	0.16	2.23*	43.2 ± 2.8	27.2 ± 1.6	−16.0 ± 0.6 (< 0.001)	0.60 (0.00)	−1.04(0.00)	−0.86(0.00)	−2,699(0.00)
30	D03	0.315	0.20	2.83	13.8 ± 4.2	7.4 ± 3.8	−6.3 ± 1.1 (< 0.001)	0.27 (0.18)	−0.12(0.64)	−0.12(0.58)	−139(0.84)
31	D03	0.315	0.17	2.19	44.7 ± 3.3	40.2 ± 2.2	−4.4 ± 0.8 (< 0.001)	0.57 (0.00)	−0.69(0.00)	−1.00(0.00)	2,240(0.10)
32	D03	0.315	0.16	2.30	23.8 ± 3.8	19.4 ± 4.6	−4.4 ± 1.1(0.003)	0.03 (0.73)	0.01 (0.95)	−0.15(0.27)	−37(0.94)
33	D03	0.315	0.17	2.29	21.9 ± 4.8	21.6 ± 6.5	−0.3 ± 1.5(0.871)	−0.16(0.41)	0.11 (0.62)	−0.48(0.08)	−881(0.01)
46	D03	0.315	0.15	1.94	32.5 ± 2.8	22.1 ± 4.1	−10.4 ± 0.9(< 0.001)	0.31 (0.07)	−0.45(0.00)	0.16 (0.47)	−601(0.14)
34	D06	0.420	0.31	2.94	30.3 ± 2.5	27.1 ± 2.4	−3.3 ± 0.7(< 0.001)	−0.00(0.96)	0.08 (0.39)	−0.77(0.00)	−591(0.00)
36	D06	0.630	0.27	3.10	32.3 ± 2.3	32.0 ± 1.8	−0.3 ± 0.6(0.694)	0.68 (0.01)	−0.18(0.41)	−0.14(0.32)	684 (0.24)
23	D20	0.795	1.03	3.91*	24.5 ± 5.3	26.3 ± 3.4	1.8 ± 1.5(0.351)	−0.02(0.92)	0.17 (0.43)	0.14 (0.53)	−488(0.34)
24	D20	1.620	0.77	6.24	35.8 ± 4.9	39.8 ± 2.1	4.0 ± 1.0(0.001)		−0.21(0.36)	−0.07(0.79)	676 (0.63)

Mean Cmax averages across individual treatments; Total AUC sums individual treatments; Δ = pre-/post-treatment change in 28-day means of: total daily symptom score (TDSS); ADL = the extent to which activities of daily living were completed; PEM = the extent to which activities were avoided for fear of inducing PEM; Exertion = the perceived level of exertion; and Steps = Fitbit^TM^-recorded level of activity (steps). TDSS data indicated as mean ± standard deviation. *Ignores leaked treatment.

Actual CT38s dose varied due to changes in individual treatments (Figure 3) and drug preparation (which added a small drawn volume of active solution to a diluent). Thus, PK parameters were calculated for individual treatments, yielding mean Cmax and total AUC (Table 2).

### CRFR2 Sensitivity

**FIGURE 4:**
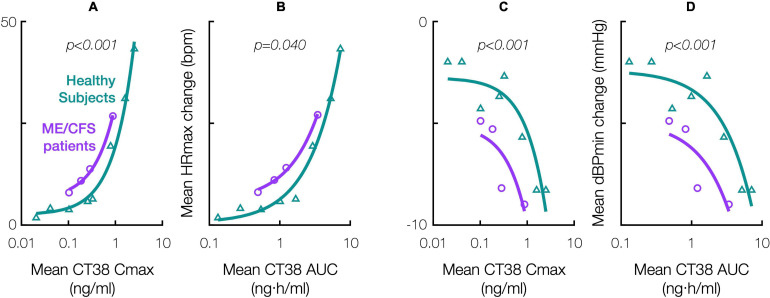
Effect of CT38 in healthy subjects (triangles, green line) and ME/CFS patients (circles, purple line), on the change from baseline in: mean maximum HR (HRmax) versus **(A)** mean Cmax or **(B)** mean AUC; and mean minimum dBP (dBPmin) versus **(C)** mean Cmax or **(D)** mean AUC; with relevant *p*-values (italics) from statistical comparisons by Kolmogorov-Smirnov test.

Relative to healthy subjects in the prior Phase 1 trial (Supplementary Material), ME/CFS patients were objectively more sensitive to the hemodynamic effects of CRFR2 stimulation during treatment. For a given CT38 concentration, the hemodynamic effects were greater in ME/CFS patients than in healthy subjects, and this differential diminished with increasing concentration, e.g., CT38 Cmax of 0.3 and 0.8 ng/ml result in mean HR increases in patients that are, respectively, 1.8× and 1.6× the corresponding change in healthy subjects (Figures 4A–D). Hemodynamic sensitivity was significantly different between ME/CFS patients and healthy controls, with their cumulative distributions for maximum hemodynamic change (HR or dBP) per unit of PK parameter (Cmax or AUC) differing between individual patient treatments (*n* = 35, InTiME) and individual healthy subject doses (*n* = 47, prior Phase 1) by Kolmogorov-Smirnov test (Figure 4).

### Efficacy

#### Intent-to-Treat Population

CT38s was associated with a statistically significant improvement in mean TDSS (TDSS_*pre*_: 29.5 ± 3.7, TDSS_*post*_: 25.3 ± 3.7, *p* = 0.011, change: −4.3 ± 1.4 or −14.5%) among all patients receiving drug (Table 2).

#### Biphasic Dose-Response

**FIGURE 5:**
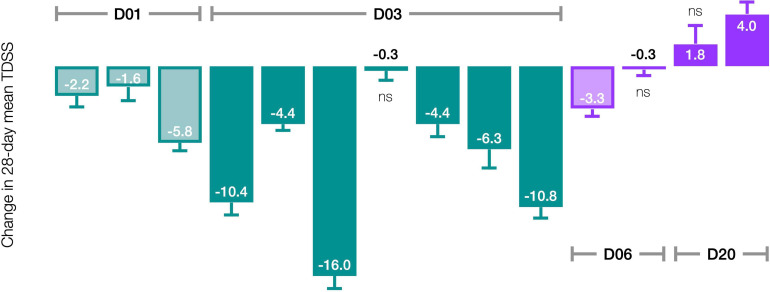
Effect of CT38 on the pre-/post-treatment change in the 28-day mean TDSS (bars), with standard deviations (error bars), by patient. All changes are statistically significant (*p* < 0.05), except ns (not significant).

The pre-/post-treatment change in 28-day mean TDSS for individual patients was statistically significant in 11 of 14 patients (Figure 5). CT38 effect appeared to be biphasic, with symptoms improving at D01 and D03, but worsening at D20. At D06, symptoms were between D03 and D20. The AUC for ID27 (D03) exceeded that of ID34 and ID36 (D06), suggesting that this biphasic character might be driven by concentration rather than AUC.

**FIGURE 6:**
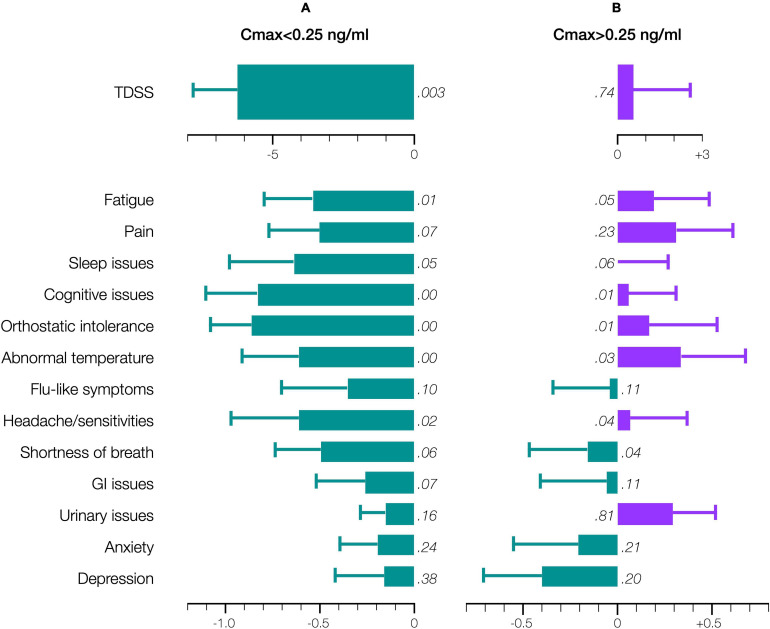
Effect of CT38 on the pre-/post-treatment change in the 28-day means (bars), with standard deviations (error bars), of TDSS and individual symptoms scores, for CT38: **(A)** Cmax < 0.25 ng/ml; or **(B)** Cmax > 0.25 ng/ml, either improving (green) or worsening (purple) with relevant *p*-values (in italics). Note that scales for TDSS and individual symptoms are different.

**FIGURE 7:**
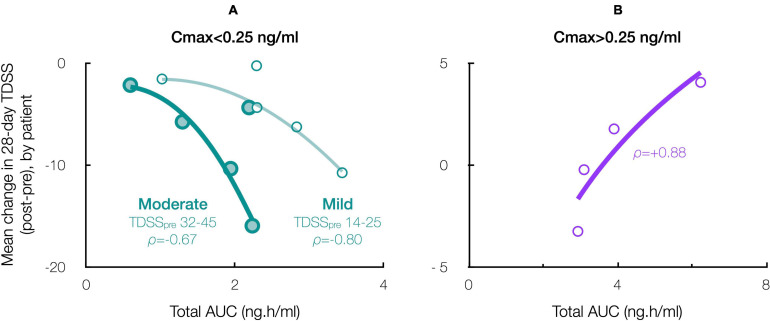
Effect of CT38 total AUC on the patient-specific change in 28-day mean TDSS, with *ρ* = Pearson’s correlation coefficient, for CT38: **(A)** Cmax < 0.25 ng/ml (green), stratified by TDSS_*pre*_ (moderate: large circles, dark line; mild: small circles, light line); or **(B)** Cmax > 0.25 ng/ml (purple).

Accordingly, stratifying for a Cmax threshold of 0.25 ng/ml (beyond which symptom improvement declined), the 28-day mean TDSS improved significantly for Cmax < 0.25 ng/ml (*n* = 10, TDSS_*pre*_: 29.1 ± 3.6, TDSS_*post*_: 22.9 ± 4.1, *p* = 0.003, change: −6.2 ± 1.6 or −21.4%), with all individual symptoms improving and several achieving significance (Figure 6A). The improvement in 28-day mean TDSS correlated directly with AUC and indirectly with pre-treatment symptom severity (Figure 7A, respective Pearson’s correlation coefficients: −0.67 and −0.80 for patients with moderate symptoms, TDSS_*pre*_ = 32–45, or mild symptoms, TDSS_*pre*_ = 14–25). For Cmax > 0.25 ng/ml, the 28-day mean TDSS worsened though not significantly (Figure 6B, *n* = 4, TDSS_*pre*_: 30.7 ± 4.0, TDSS_*post*_: 31.3 ± 2.3, *p* = 0.740, change: +0.6 ± 2.0 or +1.8%), but this lack of significance resulted from different AUCs, as there was a strong direct correlation between 28-day mean TDSS change and AUC (Figure 7B, Pearson’s correlation coefficient: +0.88).

#### SF-36

**FIGURE 8:**
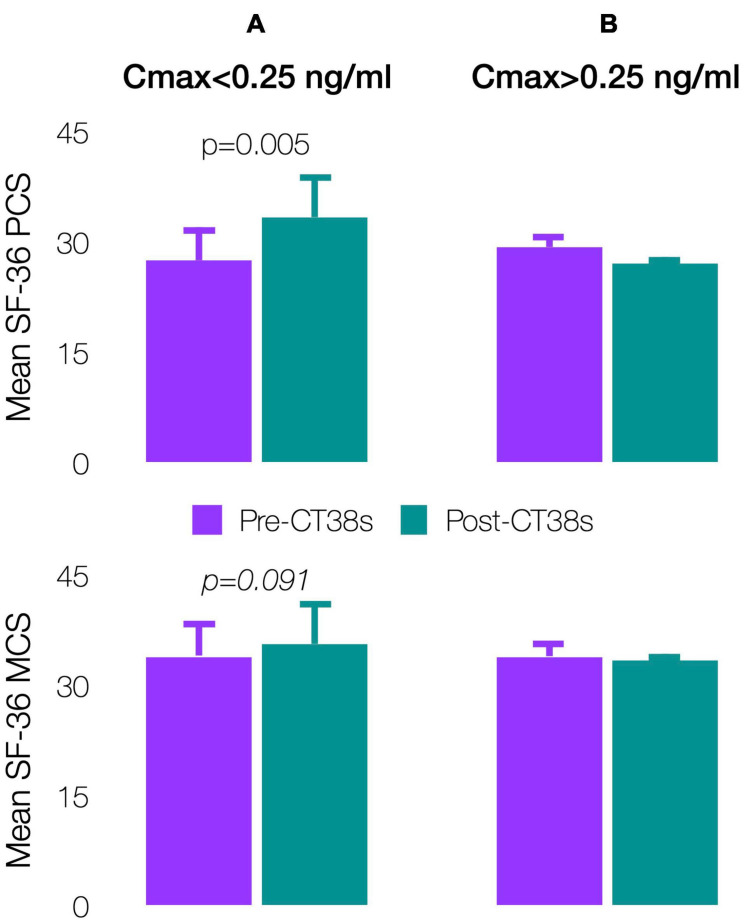
Effect of CT38 on the means of pre-treatment (purple bars) and post-treatment (green bars), with standard deviations (error bars), of SF-36 physical component score (PCS) and SF-36 mental component score (MCS) for CT38: **(A)** Cmax < 0.25 ng/ml; and **(B)** Cmax > 0.25 ng/ml.

Patients’ pre-treatment, SF-36 physical (PCS: 27.9 ± 4.0) and mental (MCS: 34.0 ± 3.6) component scores indicated worse health status than patients with cancer, congestive heart failure or diabetes (respective US means PCS/MCS: 45.1/48.8, 31.0/45.7, and 39.3/47.9) (Ware et al., 1994). The 4-week pre-treatment and pre-exit comparisons improved (significantly for PCS, *p* = 0.005) for Cmax < 0.25 ng/ml, but worsened for Cmax > 0.25 ng/ml (Figures 8A,B). Although not significant, results were substantially similar by TDSS and SF-36 (the latter being widely-validated though not in ME/CFS), so supporting the TDSS endpoint.

#### Other Endpoints

The effect of CT38s on activity was variable (Table 2). For Cmax < 0.25 ng/ml, patients completed their ADL more (pre: 1.8 ± 0.6, post: 2.0 ± 0.6, *p* = 0.078, change: +0.2 ± 0.3 or +10.0%) and avoided PEM-inducing activities less (pre: 2.2 ± 0.7, post: 2.0 ± 0.6, *p* = 0.115, change: −0.2 ± 0.3 or −10.1%), but were less active by patient-reported level of physical/mental exertion (pre: 2.4 ± 0.8, post: 2.2 ± 0.7, *p* = 0.099, change: −0.3 ± 0.3 or −10.3%) and Fitbit^TM^-recorded steps (pre: 5,065 ± 2,675, post: 4,670 ± 2,246, *p* = 0.351, change: −395 ± 1,053 or −7.8%). There was no correlation between patient-reported level of physical/mental exertion and Fitbit^TM^ recorded steps (Pearson’s correlation coefficient = 0.09 for all patients).

Subgroup analysis for sex, age, illness duration, triggers and Fitbit^TM^-recorded sleep and HR data, did not yield treatment-related insights, but this study was not powered to determine such effects.

### Safety

There were no deaths. There was one study drug discontinuation (ID35, D01) due to symptom worsening (headache, facial numbness, dyspnea, dizziness and swollen lymph nodes) in the days following treatment, which did not require intervention.

There was one SAE during ID23’s (D20) second treatment (first treatment leaked). The patient experienced tachycardia and hypotension (baseline: 92 bpm, 108/74 mmHg; peak: 125 bpm, 89/50 mmHg), recoded as two severe treatment-emergent AEs (TEAEs). The patient required rescue saline, but recovered and remained in the trial until planned exit. The SAE was reported to the FDA and IRB. It resulted from a poor prediction of the starting dose (derived from healthy animals/humans without CRFR2 upregulation), and an inadvertent continuation of dosing after the dose-stopping criteria were met. The patient also recorded severe fatigue after each treatment, so four severe TEAEs in total.

There were 161 AEs, four occurring before treatment (headache in ID30, sleep issues in ID23, hypotension in ID31, abnormal EKG in ID38). Of the remaining 157 TEAEs, four were severe (2.5% noted above), the rest being mild (124 or 79.0%) or moderate (29 or 18.5%) and resolving without intervention. Hemodynamic changes (Figure 4) and flushing during treatment, putatively CRFR2-induced vasodilation (Venkatasubramanian et al., 2013), accounted for 12 and 37 TEAEs, respectively.

## Discussion

InTiME is the first study to identify that a CRFR2-selective agonist may provide therapeutic benefit in ME/CFS patients—nine of 10 patients where Cmax did not exceed 0.25 ng/ml, showed significant, sustained TDSS improvement ranging from −1.6 to −16.0, dependent on both AUC and pre-treatment severity. This work hypothesizes that ME/CFS is caused by CRFR2 upregulation in the raphé nuclei and limbic system, observed *in vivo* (Waselus et al., 2009; Lebow et al., 2012; Wood et al., 2013), with symptoms explained by the known effects of CRFR2 activation on this subset of 5HT neurons (Waselus et al., 2005; Kirby et al., 2008; Lukkes et al., 2008). It proposes that since CRFR2 undergoes agonist-mediated endocytosis (Markovic et al., 2008, 2011; Reyes et al., 2008, 2014; Hauger et al., 2013), treatment with an agonist may downregulate membrane-bound CRFR2. The trial results support these ideas.

### Biphasic Dose-Response

InTiME sought to invoke agonist-mediated CRFR2 endocytosis, which is considered protective against overstimulation and known to increase with agonist concentration and duration of stimulation (Markovic et al., 2008, 2011; Hauger et al., 2013). The sustained symptom improvement over at least 28 days, with a peptide whose half-life is 1.5 h, suggests that endocytosis occurred, but surprisingly, only at low CT38 dose-levels (D01 and D03). Why might this be the case? The threat response is likely terminated by UCN1-mediated CRFR2 endocytosis (Markovic et al., 2008, 2011; Hauger et al., 2013). Like other G protein-coupled receptors, CRFR2 endocytosis is mediated by β-arrestin, which was thought to be recruited by activated G proteins, so requiring agonist concentrations above the threshold at which the particular agonist activates the G proteins. Recently, however, G protein-*independent* β-arrestin recruitment has been observed with another G protein-coupled receptor (Pack et al., 2018), and may even accelerate β-arrestin recruitment (Markovic et al., 2011).

This notion may explain the biphasic dose-response. CT38 displaces CRF (respective CRFR2 binding affinities: 1.1 and 44.5 nmol), and activates G proteins at a threshold concentration of ∼0.25 ng/ml, i.e., the lowest Cmax at which HR increased in healthy subjects in the prior Phase 1 trial (Figure 4A and Supplementary Material). Thus, for mean Cmax < 0.25 ng/ml (D01 and D03), G proteins did *not* activate, and symptom improvement (Figure 5) likely resulted from G protein-independent CRFR2 endocytosis, as effect was: (i) sustained long after drug clearance; (ii) AUC-dependent, consistent with endocytosis preventing overstimulation (Figure 7A); and (iii) indirectly dependent on symptom severity, also consistent with overstimulation, since if CRFR2 drives symptoms, then mild patients with only mild CRFR2 stimulation, will require relatively more treatment to achieve CRFR2 overstimulation (Figure 7A). For mean Cmax > 0.25 ng/ml, G proteins activated during treatment (early for D20; late for D06), and sustained symptom worsening (Figure 5) suggests CRFR2 upregulation (Figure 7B), arguably demonstrating PEM (Figure 6B). These data suggest that overstimulation, and resulting endocytosis, can be achieved by extended durations at concentrations below the stimulatory threshold.

### Total Dose

**FIGURE 9:**
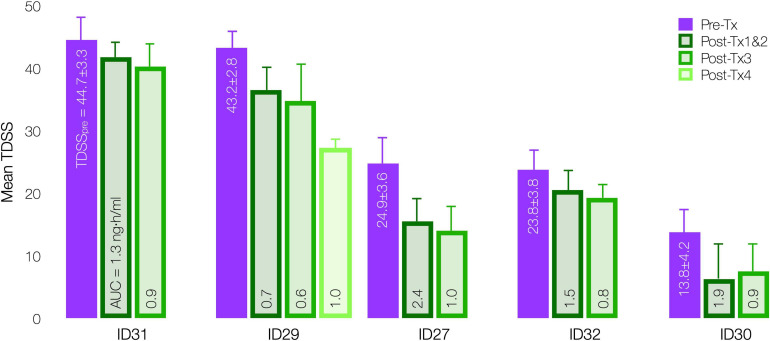
Effect of CT38 individual treatment AUC on mean TDSS pre-treatment (purple bar) and post successive treatments (green bars), with standard deviations (error bars) and the level of AUC delivered in each successive treatment, by patient.

InTiME tested whether an *acute* CT38 exposure could have a sustained effect. Tolerability and symptom worsening at concentrations above 0.25 ng/ml necessitated a reduced infusion rate, so limiting the AUC that could be delivered in the allotted timeframes. However, provided Cmax remained below 0.25 ng/ml, safety concerns were absent, and thus longer/additional infusions, to bring exposure closer to target (respectively, 3.7 and 4.9 ng h/ml for moderate and mild symptoms by extrapolation, Figure 7A), should increase efficacy. This point is evident in five patients (D03), who received 3–4 treatments sufficiently-separated to assess the effect of each treatment (Figure 9), resulting in mean TDSS decreases following treatments 1 and 2 combined (too close to separate), 3 and 4 (ID29). Overall, the see data support the notion that the AUC can be delivered as a single or multiple treatments, and increasing AUC increases effect.

### CRFR2 Sensitivity

This is the first demonstration that the CRFR2 pathway is sensitized in ME/CFS patients, so consistent with the hypothesis. As noted above, CT38 plasma concentrations below 0.25 ng/ml do not activate G proteins in healthy subjects, and thus the elevated hemodynamic response that occurred below 0.25 ng/ml in ME/CFS patients (Figures 4A,C), likely represents elevated constitutive (agonist-independent) activity, putatively due to increased receptor expression (Black et al., 2016; Berg and Clarke, 2018). This increased sensitivity to low-level CRFR2 stimulation may aid diagnosis.

### TEAEs Versus Symptoms

As CT38s is CRFR2-selective and has no off-target activity, the TEAEs resulted from CRFR2 stimulation. They included fatigue, myalgia, aches, sleep disturbance, forgetfulness, cognitive disturbance, dizziness, dysequilibrium, chills, influenza-like illness, sore throat, swollen lymph nodes, headache, paresthesia, shortness of breath, constipation, diarrhea, anxiety, emotional liability, etc. (and headache, dyspnea, sore throat and pain, in healthy subjects in the Phase 1, Supplementary Material). Such TEAEs are also known symptoms of ME/CFS. In fact, of the 108 TEAEs (excluding treatment day hemodynamics and flushing), 92 were well recognized ME/CFS symptoms and can be classified under the headings used in the TDSS endpoint (Table 3). This overlap of ME/CFS symptoms and TEAEs resulting from CRFR2 stimulation, including those in *healthy* subjects, supports the involvement of CRFR2 in ME/CFS.

**TABLE 3:** Treatment-emergent adverse events, stratified by Cmax.

	Cmax < 0.25 ng/ml (*T* = 29)		Cmax > 0.25 ng/ml (*T* = 9)			Total
	Mild	Moderate	Mild	Moderate	Severe	
Total	99	12	25	17	4	157
**During Treatment**						
Flushing	27	1	3	6		37
Cardiovascular	4	1	4	1	2	12
**TDSS-like TEAEs**						
Headaches or sensitivities	12	3	3	1		19
Fatigue	7		3	2	2	14
Flu-like symptoms	10	1	1	1		13
Temperature sensations	8	1	2			11
GI symptoms	4	2	3			9
Sleep issues	6			1		7
OI symptoms	4	1		1		6
Muscle or joint pain	3			1		4
Cognitive symptoms	3					3
Dyspnea	2	1				3
Depression				2		2
Anxiety			1			1
**Other**						
Cardiovascular		1	2	1		4
Psychological	3		2			5
Neurological	3		1			4
Pain	2					2
Metabolism or nutritional	1					1

*Data indicate actual number of recorded events; T, total number of drug treatments, by group.*

The highest InTiME dose (D20: 0.795–1.620 μg/kg, Cmax = 1.15–1.32 ng/ml, AUC = 3.91–9.85 ng h/ml) was associated with sustained effects (e.g., headache, dyspnea) in ME/CFS patients that were transient at a comparable dose in the Phase 1 healthy subjects (1.667 μg/kg, Cmax = 2.46 ng/ml, AUC = 7.11 ng h/ml, Supplementary Material). That is, where healthy subjects reversed the effects of high-dose CT38s administration, ME/CFS patients did not, suggesting an impaired ability to reverse the effects of intense CRFR2 stimulation and therefore a susceptibility to CRFR2 maladaptation.

### Long-Term Data

InTiME showed at least 28-day effect with a drug that clears in hours. Long-term follow-up in nine (of 14) patients, who are the PI’s patients (with medical chart history), show that the effects are holding over a year from treatment (close to 2 years in the earlier-treated patients). Patients noted subtle improvements in sleep, brain fog, appetite, activity and PEM (crashed less often, recovered more rapidly, but it took weeks to appreciate these changes). While anecdotal, these data support the hypothesis/treatment approach.

### Disease Pathway

*In vivo*, threat-specific CRFR2 upregulations in the raph nuclei (Lukkes et al., 2008; Waselus et al., 2009; Wood et al., 2013) and limbic system (Sananbenesi et al., 2003; Lebow et al., 2012; Elharrar et al., 2013; Qi et al., 2014) have been shown to modulate limbic 5HT, and these upregulations can persist long after threat resolution. Consistent with this, high-dose CT38 modulates functions known to be mediated by limbic 5HT in healthy rats (Supplementary Figure 1), including norepinephrine/corticosterone release (Dedic et al., 2018; Deussing and Chen, 2018; Godoy et al., 2018), spontaneous movement, possibly motor effect (Perrier et al., 2013; Perrier and Cotel, 2015), breathing (Hilaire et al., 2010), thermoregulation (Lin et al., 1998; Boulant, 2000), and HR (Oliveira et al., 2015). CT38 modulates these same functions in ME/CFS patients, exacerbating them for Cmax >0.25 ng/ml while improving them for Cmax <0.25 ng/ml, respectively, consistent with CRFR2 upregulation and endocytosis (Figure 6). This parallelism potentially positions CRFR2 in the raphé nuclei and limbic system as a pathway that responds to various environmental threats, e.g., pathogens, physical/mental trauma, chemicals, toxins (Table 1; Kerr and Mattey, 2008; Devendorf et al., 2016; Chu et al., 2019), upregulating in threat-relevant neurons (Devendorf et al., 2016), in a manner affected by prior threats/upregulations (Heim et al., 2006; Nater et al., 2011). CRFR2 can fail to downregulate, influenced by defects in threat response-related genes (Goertzel et al., 2006) and female sex (Bangasser et al., 2010, 2013; Bangasser, 2013; Howerton et al., 2014; Weathington et al., 2014; Lukkes et al., 2016), effectively changing the homeostatic set point, leading to 5HT deregulation, lost homeostasis, and the persistent signs and symptoms of ME/CFS. If validated, this pathway has several implications.

First, if CRFR1/CRFR2 are pivotal in homeostasis as proposed, the very nature of homeostatic threat dictates that CRFR1/CRFR2 adaptations are neuronally-specific, and thus maladaptations are also neuronally-specific. This ties individual signs/symptoms to specific neurons, e.g., CRFR2-induced 5HT elevations in the motor pathway could inhibit motor neuron firing (Perrier et al., 2013; Perrier and Cotel, 2015) manifesting as fatigue (Supplementary Figures 1B,C), while CRFR2-induced 5HT elevations in medullary respiratory neurons (Hilaire et al., 2010) could diminish breathing capacity (Supplementary Figure 1F). This may explain how the same symptom can present in different diseases (e.g., fatigue in ME/CFS, fibromyalgia, multiple sclerosis), and how symptoms can vary within a given disease.

Second, for limbic response to be precise, neuronal CRFR1/CRFR2 expression can *only* depend on the threat and its resolution. This suggests the absence of other influences on receptor levels, and thus treatment-induced CRFR2 endocytosis (mimicking threat resolution) should persist, thereby removing the impetus for elevated 5HT and allowing the 5HT_1A_ autoreceptors to normalize and properly inhibit 5HT. Importantly, such endocytosis does not alter the adaptive nature of the system; it only restores the set points, reducing the level of 5HT release for a given threat. Future threats will continue to modulate CRFR1/CRFR2, albeit releasing less 5HT, but nothing prevents future severe threats from provoking maladaptations that might accumulate and eventually cause dysfunction.

Third, many acquired chronic diseases (Kotas and Medzhitov, 2015; Furman et al., 2019), including ME/CFS (Nacul et al., 2020), have been theorized to involve lost homeostasis brought on by chronic low-grade inflammation, possibly arising from chronic infection, diet, gut dysbiosis, environment, etc., but explaining how such inflammation leads to specific symptoms is challenging. CRFR1/CRFR2 maladaptation offers an alternative explanation for lost homeostasis and specific symptoms. This pathway controls autonomic and endocrine function (Dedic et al., 2018; Deussing and Chen, 2018; Godoy et al., 2018), and CRFR2-selective agonists modulate metabolic activity in obesity models (Jamieson et al., 2011; Chen et al., 2013; Paruthiyil et al., 2018) and immune response in models of sepsis (Gonzalez-Rey et al., 2006a), Crohn’s disease (Gonzalez-Rey et al., 2006b), rheumatoid arthritis (Gonzalez-Rey et al., 2007) and cancer (Argilés et al., 2008), suggesting that CRFR2 plays a fundamental role. By implication, CRFR2 maladaptation could induce widespread dysfunction.

Fourth, maladaptations in limbic CRFR1/CRFR2 have no direct sequelae in bodily fluids. This is important because symptoms in the absence of bodily fluid abnormalities often lead to psychiatric diagnoses (common in ME/CFS), when the problem could be CRFR2 maladaptation. Equally, peripheral abnormalities need not imply peripheral dysfunction, e.g., poor lung function by spirometry might be diagnosed and treated as lung obstruction, yet could result from CRFR2-induced changes in respiratory rate and tidal volume (Supplementary Figure 1F). Thus, an understanding of this pathway could have important diagnostic and treatment implications.

Fifth, given the proposed connection between individual neurons and symptoms, and the overlap of triggers, signs/symptoms with those of ME/CFS, the authors postulate that the pathway and treatment approach may apply to post-acute sequelae of SARS-CoV-2, chronic Lyme disease, fibromyalgia, post-traumatic stress disorder and multiple chemical sensitivities.

In sum, the authors propose that the CRFR1/CRFR2-5HT pathway controls homeostasis and that its disruption leads to lost homeostasis. If validated, this could fundamentally alter current conceptions and treatment of many acquired chronic diseases.

### Limitations

InTiME had several limitations. It was small (*n* = 14) and open-label as treatment causes flushing. Tolerability (mild at D06, severe at D20) limited 2 dosing groups, and necessitated CT38 concentration reductions that were only partially offset by longer infusions, so target AUCs were low relative to the target dose. The concentrations of interest were close to the PK limit of quantitation (0.20 ng/ml). TDSS is not a validated endpoint in ME/CFS.

### Conclusion

This study hypothesizes that ME/CFS is caused by CRFR2 upregulation in the raphé nuclei and limbic system, and it tests agonist-mediated CRFR2 endocytosis as a novel treatment approach. The results support CRFR2 involvement in ME/CFS, and identify a treatment paradigm that is Cmax-limited and both AUC- and severity-dependent, leading to sustained symptom improvement. The PK-dependence of this response argues against a chance effect. These findings warrant further study.

## Data Availability Statement

The datasets presented in this article are not publicly available because they are part of an ongoing submission to the United States FDA. Requests to access the datasets should be directed to GP, gpereira@corteneinc.com.

## Ethics Statement

The studies involving human participants were reviewed and approved by Aspire Institutional Review Board and Independent Investigational Review Board Inc. The patients/participants provided their written informed consent to participate in this study. The animal studies were reviewed and approved by P&G’s Institutional Animal Care and Use Committee and/or attending veterinarian in full compliance with the Animal Welfare Act.

## Author Contributions

GP conceived the hypothesis and treatment approach, and drafted the article. All authors developed the protocol. LB and HG served as the study PI and Medical Monitor, respectively. GP, HG, SC, and MC proposed dosing and changes thereto, which LB approved. LB, TM, and SV collected the data. MC and GP assembled and analyzed the data. All authors revised and approved the final version.

## Conflict of Interest

GP, HG, SC, MC, and LB are shareholders of Cortene Inc., which owns the commercial rights to the drug being tested. The remaining authors declare that the research was conducted in the absence of any commercial or financial relationships that could be construed as a potential conflict of interest. The authors declare that this study received funding from Cortene, Inc. The funder conceived of the idea to test CT38 in ME/CFS and was involved in the overall study design, protocol preparation, data analysis and writing the manuscript. The funder was not involved in recruiting patients, data collection or the conduct of the clinical trial.

## Publisher’s Note

All claims expressed in this article are solely those of the authors and do not necessarily represent those of their affiliated organizations, or those of the publisher, the editors and the reviewers. Any product that may be evaluated in this article, or claim that may be made by its manufacturer, is not guaranteed or endorsed by the publisher.

## Abbreviations

5HT: serotonin
5HT_1A_: 5HT receptor type 1A
ADL: activities of daily living
AE: adverse event
AUC: area under the plasma concentration-time curve
BNST: bed nucleus of the stria terminalis
Cmax: maximum plasma concentration
CPET: cardio-pulmonary exercise test
CRF: corticotropin-releasing factor
CRFR1/2: CRF receptor type 1/2
dBP: diastolic blood pressure
FDA: United States Food and Drug Administration
GABA: gamma-aminobutyric acid
HR: heart rate
MAP: mean arterial pressure
MCS: SF-36 mental component score
ME/CFS: myalgic encephalomyelitis/chronic fatigue syndrome
OI: orthostatic intolerance
PCS: SF-36 physical component score
PEM: post-exertional malaise
PI: principal investigator
PK: pharmacokinetic
SAE: serious AE
sBP: systolic blood pressure
SF-36: 36-Item Short Form Survey Instrument
SSRI: selective serotonin reuptake inhibitor
TDSS: total daily symptom score
TEAE: treatment-emergent AE
UCN1/2/3: urocortin 1/2/3.
