# Supplementary material for: Acute Corticotropin-Releasing Factor Receptor Type 2 Agonism Results in Sustained Symptom Improvement in Myalgic Encephalomyelitis/Chronic Fatigue Syndrome
Source: Front Syst Neurosci. 2021 Sep 1;15:698240. doi: 10.3389/fnsys.2021.698240 (PMC8441022; doi:10.3389/fnsys.2021.698240)
Supplement: Supplementary file 1 [file Data_Sheet_1.pdf]

frontiers

Supplementary Material

CT38s (acetate salt of CT38 free base) is being developed as a therapeutic for a number of indications. The data included here are a subset of a comprehensive body of work including *in vitro* and *in vivo* studies and a prior Phase 1 clinical trial in healthy subjects, all submitted to the FDA

CT38 *In Vitro* Studies

CRFR1 and CRFR2 selectivity and potency studies (ID M020-1311) assessing CT38, CT38s, CRF, UCN1, UCN2, UCN3 and numerous other CRFR2 agonists, utilized CRFR1-containing (CRFR2-lacking) human retinoblastoma Y-79 cell line (ATCC HTB-18, cultured in RPMI 1640/10% fetal bovine serum media, Life Technologies Inc., Rockville MD), and CRFR2-containing (CRFR1-lacking) rat aortic cell line A7r5 (ATCC CRL1444, cultured in DMEM/10% fetal bovine serum medium, Life Technologies Inc., Rockville MD). Cells were plated into 96-well plates at 20,000 cells/well: Y-79 cells (suspension culture;) the morning of the assay in 90 µL serum-free DMEM containing 2.5mM IBMX (SIGMA-Aldrich, St. Louis, MO); A7r5 cells (adherent culture) 24 hours prior to assay in 100 µL of typical growth media, and removed from media and washed with PBS and then incubated in 90 µL serum-free DMEM containing 2.5 mM IBMX. Following 30 minutes of incubation at 37°C with the test compound (10 µL of compound added at 10x final indicated concentrations), the media was aspirated off the cells (Y-79 cells were spun at 1000 rpm for 5 minutes in a tabletop centrifuge), the cells were lysed in lysis buffer (Amersham RPN225), and intracellular cAMP levels were quantified using an RIA kit (Amersham Bioscience Corp., Piscataway, NJRPN225) per manufacturer-recommended procedures. Potencies, EC50, and Emax calculations were performed using Graph Pad Prism software; EC50 data were analyzed using a non-linear regression, sigmoidal dose-response (variable slope) equation. Resulting EC50 (nmol)/percent of Emax (%) at CRFR1 and CRFR2 respectively, were: CT38: >1,000/53 and 17.1/100; CT38s: >1,000/72 and 10.0/94; CRF: 20.0/87 and 49.3/82.3; UCN1: 9.0/100 and 3.5/100; and UCN2: >1,000/7.6 and 4.3/96. The inhibitory constant for CT38 at CRFR2 was estimated to be 1.1 nmol, similar to that of UCN2.

Binding affinity studies (ID 53101) evaluated CT38 in a large pharmacological profile including 85 receptors, transporters, ion channels and enzymes (Cerep, Poitiers, France), determining the inhibition of specific radioligand binding or enzyme activity by CT38. At a concentration of 10 µM, CT38 displayed affinity for CRFR1 (71% inhibition) and little or no affinity for the others receptors, transporters, ion channels and enzymes studied, specifically including receptors and transporters for serotonin ($5HT_{1A}$, $5HT_{2A}$, $5HT_3$, 5HTT), norepinephrine/epinephrine (α_{2A}, β_1, β_2), GABA ($GABA_A$, $GABA_B$), and glucocorticoids (GR).

CT38 *In Vivo* Studies

The effect of CT38s (and CT38) on stress hormone levels, spontaneous movement (controlled by the central nervous system), and respiratory, gastrointestinal, urinary and hemodynamic function, was investigated in rats. These studies utilized doses based on prior evaluations and/or were intended to

demonstrate an effect gradient. The analysis used a one-way ANOVA to identify dose-dependent effects of CT38 on each measured endpoint. All studies were approved by P&G's Institutional Animal Care and Use Committee and/or attending veterinarian and complied with the Animal Welfare Act. The results are shown as Supplementary Figure 1A-I and Supplementary Figure 2A-D.

Stress hormones study (ID 53879): 12 male Sprague-Dawley rats, with indwelling jugular cannula (acquired from Charles River Labs, 8-9 weeks old, weighing 314-340 g), were acclimated (over 1 day) to 12 hour light/dark cycles in cages (1/cage) with ad libitum access to food/water. CT38 (vehicle: 0.05 M TRIS, 0.1% EDTA, 0.55% NaCl at pH 7.0 to 7.4) was administered by single subcutaneous bolus at varying dose-levels (0 and 1,000 µg/kg, 6 rats/dose), followed by venous blood

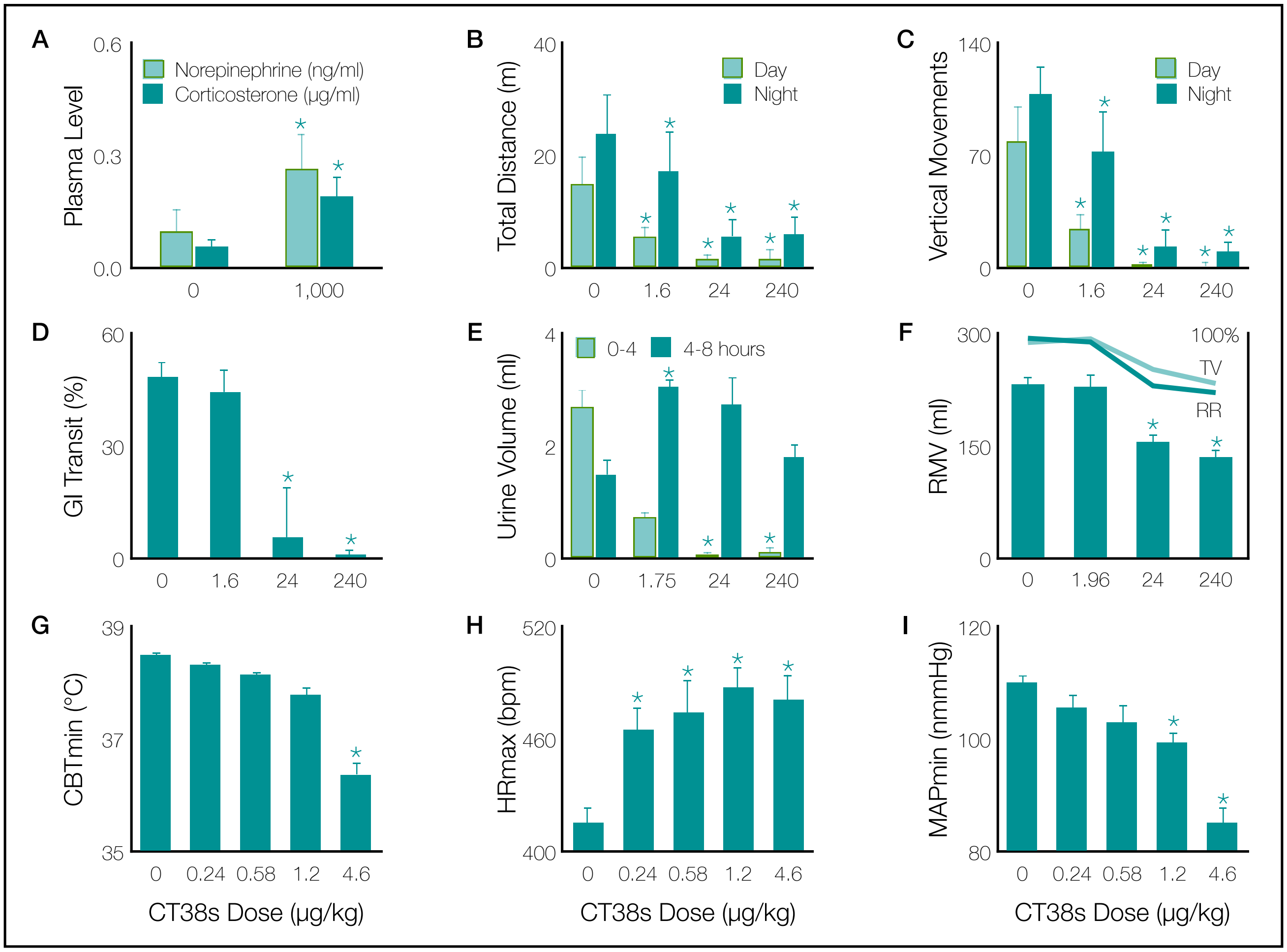

Supplementary Figure 1: Effect in rats of subcutaneous bolus doses of CT38 (µg/kg) or vehicle (0 µg/kg), on mean (bar), standard deviation (error bar) and 2-sided t-test statistical significance (*p≤0.05) for: (**A**) norepinephrine (ng/ml, light green) and corticosterone (µg/ml, dark green), 2 hours post-dose; (**B**) total distance moved in 20 mins during day (light green) or night (dark green), commencing 10 mins post-dose; (**C**) number of vertical movements in 20 mins during day (light green) or night (dark green), commencing 10 mins post-dose; (**D**) gastrointestinal (GI) transit time, 1 hour post-dose; (**E**) urine volume at 0-4 hour (light green) and 4-8 hours (dark green) post-dose; (**F**) respiratory minute-volume (RMV), via change from baseline (%) in respiratory rate (RR, dark green line) and tidal volume (TV, light green line), 1 hour post-dose; (**G**) minimum core body temperature (CBTmin), over 24 hours post-dose; (**H**) maximum heart rate (HRmax), over 24 hours post-dose; and (**I**) minimum mean arterial pressure (MAPmin), over 24 hours post-dose.

collection at 2 and 4 hours post-dose. Samples were processed and shipped to analytical laboratory (PPD Discovery) for determination of levels of epinephrine, norepinephrine, corticosterone and aldosterone (via purpose developed and validated liquid chromatography tandem mass spectrometry assays).

Spontaneous movement study (ID 54311): 16 male Sprague-Dawley rats (acquired from Charles River Labs, 7 weeks old, weighing 188-285 g), were acclimated (over 16 days) to 12 hour light/dark cycles in cages (1/cage) with ad libitum access to food/water. CT38s (vehicle: 0.05 M TRIS buffer, 0.67% NaCl in sterile H_2O for injection, USP, pH 7.5-7.7) was administered by single subcutaneous bolus at varying dose-levels (0, 1.6, 24, 240 µg/kg, with each rat receiving all doses in a Latin square with ≥45 hour washout between doses, i.e., 8 rats/dose for day and 8 rats/dose for night). Following dosing, the Accuscan VersaMax Animal Activity Monitoring System was used to track distance moved (m) and number of vertical movements in a 20-minute period commencing 10 mins post-dose.

Gastrointestinal study (ID 54473): 32 male Sprague-Dawley rats (acquired from Charles River Labs, 7-8 weeks old, weighing 188-285 g), were acclimated (over 3 days) to 12 hour light/dark cycles in cages (1/cage) with ad libitum access to food/water. Following removal of food/water, CT38s (vehicle: 0.05 M TRIS buffer, 0.67% NaCl in sterile H_2O for injection, USP, pH 7.5-7.7) was administered by single subcutaneous bolus at varying dose-levels (0, 2.4, 24, and 240 µg/kg, 8 rats/dose randomized). Rats were given a charcoal meal (oral gavage) at 30 mins post-dose, euthanized (CO_2 inhalation) 1 hour post-dose, and the charcoal distance traveled was measured (as a percent of intestinal length).

Urinary study (ID 54315): 32 male Sprague-Dawley rats (acquired from Harlan Teklad, 7 weeks old, weighing 189-213 g), were acclimated (over 3 days) to 12 hour light/dark cycles in cages (4/cage) with ad libitum access to food/water. On the treatment day, food/water were removed, and 2 hours later, rats were given sterile saline (20 ml/kg oral loading), following which CT38s (vehicle: 0.05 M TRIS buffer, 0.67% NaCl in sterile H_2O for injection, USP, pH 7.5-7.7) was administered by single subcutaneous bolus at varying dose-levels (0, 1.75, 24, and 240 µg/kg, 8 rats/dose randomized). Rats were placed in metabolic cages (1/cage, without access to food/water for 8 hours), and urine samples were collected at 4, 8, and 24 hours post-dose for subsequent determination of urine volume and chemistry.

Respiratory study (ID 54313): 32 male Sprague-Dawley rats (acquired from Harlan Teklad, 8 weeks old, weighing 212-280 g), were acclimated (over 6 days) to 12 hour light/dark cycles in cages (4/cage) with ad libitum access to food/water. Rats were acclimated to the plethysmography chambers on 2 occasions for 15-min periods, and then again on the test day, when pre-dose respiration rate and tidal volume was acquired (using the Notocord HEM Version 3.4.0.42 data capture system). Following removal from the chamber, CT38s (vehicle: 0.05 M TRIS buffer, 0.67% NaCl in sterile H_2O for injection, USP, pH 7.5-7.7) was administered by single subcutaneous bolus at varying dose-levels (0, 1.96, 24, and 240 µg/kg, 8 rats/dose randomized). Rats were returned to the chamber for 15-min periods to record respiration rate and tidal volume at 1 and 2 hours post-dose.

Hemodynamic studies (IDs 54795, 53144, 56569 and 53180): Sprague-Dawley rats (acquired from Charles River Labs), aged 6-13, 20, 6-7 and 20 weeks (by study, respectively), weighing 200-400, 180-300, 275-375 and 200-250 g (by study, respectively) were acclimated over 3, 12, 3 and 12 days (by study, respectively), to 12 hour light/dark cycles in cages (1/cage) with ad libitum access to food/water. Anesthetized rats were implanted with a TL11M2-C50-PXT telemetry device (capable of

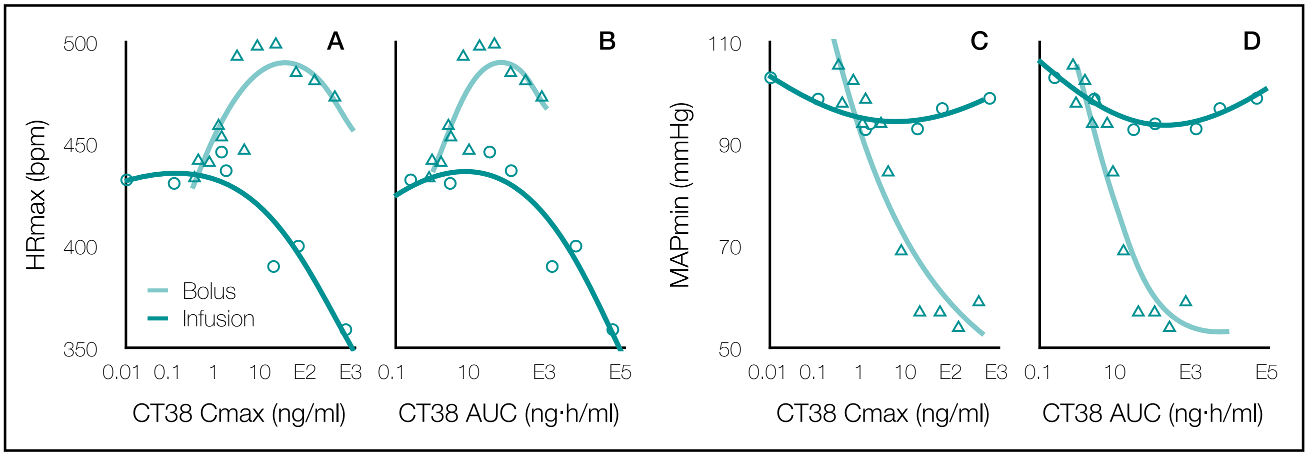

Supplementary Figure 2: Effect in rats of subcutaneous CT38, delivered by bolus (triangles, light line) or infusion (circles, dark line), on the means of: maximum heart rate (HRmax) with (**A**) Cmax or (**B**) AUC; and minimum mean arterial pressure (MAPmin) with (**C**) Cmax or (**D**) AUC.

measuring HR, blood pressure and core body temperature) at least 1 week pre-dose (all studies); subcutaneous dosing catheters were inserted in the rats 1 day (ID 56569) and 3 days (ID 53180) pre-dose. CT38 in vehicle (distilled H_2O; TRIS/EDTA buffer at pH 7.4; TRIS NaCl buffered at pH 7.4-7.6 containing 0.1% Tween 80; distilled H_2O; by study, respectively) was administered, either by single subcutaneous bolus at varying dose levels (0, 0.24, 0.58, 1.2 and 4.6 µg/kg, with each rat receiving all doses in a Latin square with ≥60 hour washout between doses, 16 rats/dose in ID 54795); or by single subcutaneous bolus at varying dose levels (0, 0.3, 1, 3, 10, 30, 100, 300 µg/kg, with each rat receiving all doses in a Latin square with ≥48 hour washout between doses, 6 rats/dose in ID 53144); or by 24-hour subcutaneous infusion at varying dose-levels (0, 0.24, 2.4, 24 µg/kg/day, with each rat receiving all doses in a Latin square with ≥48 hour washout between doses, 16 rats/dose in ID 56569); or by 72-hour subcutaneous infusion at varying dose-levels (0, 30, 300, 1,000, 10,000 µg/kg/day, with each rat receiving all doses sequentially with no washout between doses, 6 rats/dose in ID 53180). The telemetry device recorded heart rate (bpm), mean arterial blood pressure (mmHg) and core body temperature (°C) continuously from 2 hours pre-dose to 24 hours post-dose.

PK evaluations established the time to maximum plasma concentration (tmax=0.57 hours) and half-life ($t_{1/2}$=0.89 hours) for CT38s in rats.

Phase 1 Clinical Trial

A randomized, placebo-controlled, single-ascending dose Phase 1 trial was conducted at Seaview Research (Miami, FL), according to ICH/GCP, under a protocol approved by the FDA and Independent Investigational Review Board Inc (Plantation, FL), with each subject providing signed informed consent. In the study, CT38s (vehicle/placebo: 0.05 M TRIS buffer, 0.67% NaCl in sterile H_2O for injection, USP, pH 7.5-7.7) was administered by subcutaneous bolus, in single ascending doses (0.004, 0.012, 0.033, 0.083, 0.200, 0.417, 0.833, 1.667 µg/kg), to establish the safety, tolerability and PK of CT38s in healthy human subjects. The trial enrolled 64 males (mean age 34.1±7.6 years; range 18-45 years) into 8 sequential cohorts, each 8 subjects (randomized 3:1), assessing HR, sBP, dBP, and PK, monitored before and at regular intervals (to 96 hours post-dose), as

well as blood tests (standard chemistry panel), urine samples (as CT38 is eliminated by renal filtration), and AEs.

There were no SAEs. There was one study voluntary discontinuation, at the highest dose. AEs were generally mild to moderate and resolved without intervention. Significant adverse events included mild to moderate flushing (21 subjects, at doses≥0.200 µg/kg); tachycardia occurring within 1 hour post-dose and resolving within 2-6 hours (5 subjects, at doses≥0.833 µg/kg); and moderate hypotension (2 subjects, at dose=1.667 µg/kg). There were no significant differences in laboratory test results, except white blood cell counts at 6 hours post-dose (at doses≥0.833 µg/kg), which had resolved by the next day.

The PK data (measured in terms of the free base, CT38) showed rapid absorption with median time to maximum concentration (tmax) occurring about 1-1.5 hours post-dose across the various dose levels. CT38 plasma concentrations decreased rapidly after tmax, with mean terminal half-life ($t_{1/2,z}$) of 1.5 hours. CT38 peak concentration (Cmax) and total exposure (AUC) increased with dose. The lowest dose at which the HR increase was statistically different from placebo was 0.083 µg/kg (mean Cmax=0.25±0.05 ng/ml, mean AUC=0.17±0.12 ng·h/ml). The lowest dose associated with a dBP decrease was 1.667 µg/kg (mean Cmax=2.46±1.51 ng/ml, mean AUC=7.11±4.02 ng·h/ml), which was not well tolerated. The maximum tolerated does was 0.833 µg/kg (mean Cmax=1.56±0.45 ng/ml, mean AUC=5.18±1.19 ng·h/ml). All hemodynamic parameters normalized within hours.
